# Supplementary material for: Inoculation with Pseudomonas spp. in Solanum lycopersicum increases yield and fruit quality under nutrient shortage conditions
Source: PeerJ. 2025 Aug 21;13:e19796. doi: 10.7717/peerj.19796 (PMC12375298; doi:10.7717/peerj.19796)
Supplement: Supplemental Information 7 [file peerj-13-19796-s007.pdf]

INFORME DE RESULTADOS

No. de Informe: **LABORATORIO**
Fecha de emisión: **20/02/2025**

Datos del cliente

Nombre del cliente: **ALFONSO LUNA CRUZ**
Dirección del cliente: **NO INDICADO**
Contacto del cliente: **ALFONSO LUNA CRUZ** Correo: **AFONSO.LUNA@UMICH.MX** Teléfono: **5951075629**

Descripción de la muestra

Identificación o lote:	**INVERNADERO**	Fecha de recepción:	**7/2/2025**	Cultivo actual:	**NO INDICADO**
Cantidad:	**1k**	Fecha de muestreo:	**NO INDICADO**	Profundidad de muestreo:	**NO INDICADO**
Tipo de muestra:	**SUELO (FISICOQUÍMICO)**	Observaciones:	**NO INDICADO**	Cultivo anterior:	**NA**

Condiciones ambientales

Lugar donde se realiza las pruebas de ensayo: **Laboratorio no. 9**

Método/prueba

pH y C.E; método electrométrico; Nitrógeno nítrico: método colorimétrico (como nitratos), carbonato y bicarbonatos: titulación; cloruros: titulación Mohr; sulfatos; método turbidimétrico; fósforo; Microelementos y cationes directos con AAS.
Castellanos, J.Z, J.X. Uvalle - Bueno y A. Aguilar-Santelisese. 2000. Manual de interpretación de análisis de suelo y aguas. Instituto de Capacitación para la Productividad Agricola. Colección INCAPA. Celaya, Guanajuato, México.

Fecha de ejecución del ensayo del 07/02/2025 al 20/02/2025

Resultado

Identificación de la muestra: **INVERNADERO**

Parámetros Físicos	Resultado	Unidades	Nivel en muestra (Muy bajo / Bajo / Mod. Bajo / Medio / Mod. Alto / Alto / Muy Alto)
pH (1:2 agua destilada)	**7.65**	NA	
Conductividad Eléctrica en Extracto de Saturación	**1.67**	mS/cm	
Materia Orgánica	**1.23**	%	
Textura (Triángulo de Texturas)	**Franco -Arenoso**	NA	
Arcilla	**15.60**	%	
Arena	**57.12**	%	
Limo	**27.28**	%	
Densidad Aparente del Suelo	**0.96**	g/cm3	
Nitrógeno Nítrico	**23.28**	mg/kg	

Aniones (-)	Resultado	Unidades	Nivel en muestra (Muy bajo / Bajo / Mod. Bajo / Medio / Mod. Alto / Alto / Muy Alto)
Carbonatos (CO_3^{2-})(mg/L extracto de saturación)	**1.81**	mg/L	
Bicarbonatos(HCO_3^-)(mg/L extracto de saturación)	**386.15**	mg/L	
Cloruros (Cl^-) (mg/L extracto de saturación)	**58.24**	mg/L	
Azufre (S)	**8.26**	mg/L	
Fósforo Olsen	**11.20**	mg/kg	

INFORME DE RESULTADOS

No. de Informe: **LABORATORIO**

Fecha de emisión: **20/02/2025**

Cationes (+)	mg/kg	Cmol/kg	% de base saturada	Nivel en muestra
				Muy bajo · Bajo · Mod. Bajo · Medio · Mod. Alto · Alto · Muy Alto
Sodio (Na^{+})	**5.34**	**0.023**	**1.41**	
Potasio (K^{+})	**42.57**	**0.109**	**6.61**	
Calcio ($Ca2^{+}$)	**296.17**	**1.478**	**89.77**	
Magnesio (Mg^{2+})	**4.41**	**0.036**	**2.20**	
Capacidad de Intercambio Catiónico		**1.646**	**100.00**	

Microelementos	Resultado	Unidades	Nivel en muestra
			Muy bajo · Bajo · Mod. Bajo · Medio · Mod. Alto · Alto · Muy Alto
Fierro (Fe^{2+})	**10.76**	mg/kg	
Zinc (Zn^{2+})	**<0.25**	mg/kg	
Cobre (Cu^{2+})	**0.52**	mg/kg	
Manganeso (Mn^{2+})	**15.76**	mg/kg	
Boro (B^{3+})	**3.01**	mg/kg	

Observaciones:

Información para el cliente

1. Con base en los resultados obtenidos, es responsabilidad del usuario el analizar los resultados de las pruebas de ensayo con respecto a las especificaciones establecidas por el mismo, para determinar el criterio de aceptación o rechazo.
2. Los resultados del presente informe solo son aplicables a la muestra descrita en este documento.
3. Este documento es válido solo en su forma original (impreso o electrónico) con las correspondientes firmas. No se debe reproducir este Informe de pruebas de ensayo, excepto en su totalidad, sin la aprobación escrita del laboratorio.
4. El Laboratorio no se hace responsable del muestreo.
5. Alcance no acreditado.

Realizó análisis

Jorge Alberto Aguilera Martinez
Asistente técnico

Anexos

Sin anexos
